# Rhythm vs. Rate Control in Patients with Postoperative Atrial Fibrillation after Cardiac Surgery: A Systematic Review and Meta-Analysis

**DOI:** 10.3390/jcm12134534

**Published:** 2023-07-07

**Authors:** Muneeb Ahmed, Emilie P. Belley-Coté, Yuan Qiu, Peter Belesiotis, Brendan Tao, Alex Wolf, Hargun Kaur, Alex Ibrahim, Jorge A. Wong, Michael K. Wang, Jeff S. Healey, David Conen, Philip James Devereaux, Richard P. Whitlock, William F. Mcintyre

**Affiliations:** 1 Faculty of Health Sciences, McMaster University, Hamilton, ON L8L 2X2, Canada; 2 Ottawa Heart Institute, University of Ottawa, Ottawa, ON K1Y 4W7, Canada; 3 Department of Medicine, University of British Columbia, Vancouver, BC V6T 1Z1, Canada; 4 Department of Medicine, Western University, Hamilton, ON N6A 5C1, Canada

**Keywords:** rhythm control, rate control, atrial fibrillation, cardiac surgery, length of stay

## Abstract

Background: Postoperative atrial fibrillation (POAF) is the most common complication after cardiac surgery; it is associated with morbidity and mortality. We undertook this review to compare the effects of rhythm vs. rate control in this population. Methods: We searched MEDLINE, Embase and CENTRAL to March 2023. We included randomized trials and observational studies comparing rhythm to rate control in cardiac surgery patients with POAF. We used a random-effects model to meta-analyze data and rated the quality of evidence using GRADE. Results: From 8,110 citations, we identified 8 randomized trials (990 patients). Drug regimens used for rhythm control included amiodarone in four trials, other class III anti-arrhythmics in one trial, class I anti-arrhythmics in four trials and either a class I or III anti-arrhythmic in one trial. Rhythm control compared to rate control did not result in a significant difference in length of stay (mean difference −0.8 days; 95% CI −3.0 to +1.4, I^2^ = 97%), AF recurrence within 1 week (130 events; risk ratio [RR] 1.1; 95%CI 0.6–1.9, I^2^ = 54%), AF recurrence up to 1 month (37 events; RR 0.9; 95%CI 0.5–1.8, I^2^ = 0%), AF recurrence up to 3 months (10 events; RR 1.0; 95%CI 0.3–3.4, I^2^ = 0%) or mortality (25 events; RR 1.6; 95%CI 0.7–3.5, I^2^ = 0%). Effect measures from seven observational studies (1428 patients) did not differ appreciably from those in randomized trials. Conclusions: Although atrial fibrillation is common after cardiac surgery, limited low-quality data guide its management. Limited available evidence suggests no clear advantage to either rhythm or rate control. A large-scale randomized trial is needed to inform this important clinical question.

## 1. Introduction

Annually, over half a million adults undergo cardiac surgery in North America [1]. These numbers are expected to increase as the global burden of cardiovascular disease grows [2,3]. Atrial fibrillation (AF) is the most common complication after cardiac surgery; postoperative AF (POAF) occurs in up to 40% of patients [4]. Patients who experience POAF are more likely to have adverse events, including up to a fourfold increase in the odds of stroke and a doubling in the odds of death [4,5,6]. Patients with POAF spend, on average, an additional 48 h in the intensive care unit, 3 more days in the hospital, and have a 30% greater chance of hospital readmission in the 30 days after surgery [5,6,7,8].

Two strategies are used to manage POAF: rhythm and rate control. Rhythm control focuses on restoring sinus rhythm with anti-arrhythmic drugs (most commonly amiodarone) or electrical cardioversion. Rate control uses one or more negative chronotropic drugs to control ventricular rate. The optimal strategy remains unclear [9,10,11]. Guidelines issued by the Canadian Cardiovascular Society (CCS), the European Society of Cardiology (ESC), the Cardiac Society of Australia and New Zealand, and the European Association for Cardio-Thoracic Surgery (EACTS) have all addressed the issue of rhythm vs. rate control after cardiac surgery, with differing conclusions [9,10,11,12].

This systematic review and meta-analysis aimed to synthesize all of the evidence (randomized trials and observational studies) on the safety and efficacy of a rhythm control strategy as compared to a rate control strategy in adult patients without a history of AF who developed POAF after cardiac surgery.

## 2. Materials and Methods

We registered the protocol with PROSPERO (2021 CRD42021259249). Supplementary File S1 lists the differences between the registered protocol and the final manuscript. This systematic review adheres to Preferred Reporting Items for Systematic Reviews and Meta-Analyses (PRISMA) guidelines [13].

### 2.1. Eligibility Criteria

We searched for published randomized trials and observational studies comparing a rhythm control to a rate control strategy in cardiac surgery patients who developed POAF after cardiac surgery. We included studies if they reported at least one of the predetermined outcomes of interest. Rhythm control was defined by the use of an anti-arrhythmic drug (i.e., a class I or III agent, including amiodarone) or electrical cardioversion, irrespective of the use of rate-controlling agents. Rate control was defined as a strategy based on any of beta blockers, non-dihydropyridine calcium channel blockers or digoxin. We did not place restrictions on language and considered both full texts and studies published only as abstracts.

### 2.2. Search Methods

We searched MEDLINE, Embase and CENTRAL from inception to March 2023. We also screened trial registries and contacted experts to identify additional studies. We designed a search strategy and reviewed it with a librarian to capture pharmacologic rhythm control with a class I or III anti-arrhythmic agent, electrical cardioversion, and pharmacologic rate control with beta blockers, non-dihydropyridine calcium channel blockers or digoxin. We present the search strategy in Supplementary File S2.

### 2.3. Selection of Studies

We selected studies using Covidence Systematic review software (Veritas Health Innovation, Melbourne, VIC, Australia). Two reviewers screened titles and abstracts independently and in duplicate and retrieved full-text reports for all items deemed potentially relevant by either reviewer. Subsequently, two authors independently compared full-text reports against eligibility criteria. We resolved any disagreements through discussion with the senior author.

### 2.4. Data Extraction

We abstracted descriptive data (e.g., patient population, intervention, comparator) from selected studies. Outcomes of interest were length of the index hospital stay, hospital readmission, new or worsening heart failure, days out of hospital, quality of life, freedom from AF (within 1 week, up to 1 month, and up to 3 months), bleeding, myocardial infarction, mortality, and stroke. We used studies’ definitions for clinical outcomes. Two reviewers independently and in duplicate extracted data using pre-designed data collection forms. We resolved disagreements through discussion with the senior author.

### 2.5. Risk of Bias

We assessed risk of bias in randomized trials using the Cochrane Risk of Bias 2 (RoB 2) tool [14]. We independently assessed the following domains in duplicate: (i) random sequence generation; (ii) allocation concealment; (iii) blinding of study participants, personnel, and outcome assessors; (iv) incomplete outcome data (we considered ≥ 20% missing data at high risk of bias); and (v) performance bias. We compared the assessments and discussed them to resolve disagreements. For analysis and presentation purposes, we dichotomized risk of bias as high (or likely high) or low (or likely low). We categorized a trial as high risk of bias if it was at risk of selection, performance, detection, or reporting bias for that outcome.

We assessed risk of bias in observational studies using the Cochrane-endorsed CLARITY tool [15]. We rated the risk of bias in studies as low, moderate, serious, or critical across seven domains: (i) bias due to confounding; (ii) selection of patients into the study; (iii) classification of the intervention; (iv) bias due to deviations from the intended intervention; (v) missing data; (vi) measurement of outcomes; and (vii) selection of reported results [15].

### 2.6. Statistical Analysis

We analyzed randomized trials and observational studies separately. We used mean difference (MD) as the standard measure of association for length of the index hospital stay and risk ratios (RRs) for all other clinical outcomes. We present 95% confidence intervals (CI) around estimates of effect. We assessed clinical and methodological heterogeneity based on study characteristics. We transformed the median and a measure of dispersion to mean and standard deviation for our meta-analyses, assuming a normal distribution [16]. We measured statistical heterogeneity using the I^2^ statistic. We considered an I^2^ greater than 50% as showing substantial heterogeneity [17]. We used RevMan 5.3 (The Cochrane Collaboration, Denmark) to combine data quantitatively. We decided a priori to use a random-effects model with Mantel–Haenszel weighting because it is conservative, and we expected clinical and methodological heterogeneity. We analyzed according to the participant’s first assigned group (intention-to-treat principle) in randomized trials where participants crossed over to the other treatment. We considered two-sided *p*-values < 0.05 to be statistically significant.

We performed pre-specified subgroup analyses comparing studies in which participants received amiodarone-based rhythm control to those in which they received other regimens (Supplementary File S3). We evaluated for interaction between subgroups and treatment effect and reported *p*-values.

### 2.7. Quality Assessment

We assessed the quality of evidence using the GRADE (Grading of Recommendations Assessment, Development and Evaluation) approach [18]. We appraised our confidence in the estimates of effects by considering risk of bias in individual studies, directness of the evidence, precision of effect estimates for individual clinical outcomes, heterogeneity of the data and potential for publication bias.

## 3. Results

### 3.1. Selection of Included Studies

From 8110 citations, we identified eight randomized trials that evaluated 10 different rhythm control regimens and included a total of 990 patients [19,20,21,22,23,24,25,26]. Table 1 outlines the characteristics of the included trials. Supplementary File S4 outlines the study selection process. Drug regimens used for rhythm control included amiodarone in four trials, other class III anti-arrhythmics in one trial, class I anti-arrhythmics in four trials and either a class I or III anti-arrhythmic in one trial. For rate control, four trials permitted choice between beta blockers, calcium channel blockers or digoxin, one trial allowed choice between calcium channel blockers or digoxin, one trial used beta blockers alone, one trial used calcium channel blockers alone and one trial used digoxin alone. Supplementary file S5 describes the rhythm monitoring methods that were used in each trial.

We identified seven observational studies that included a total of 1428 patients (Supplementary File S6) [27,28,29,30,31,32,33]. For rhythm control, five studies used amiodarone while two studies permitted choice between a class I or III anti-arrhythmic. For rate control, two studies allowed choice between beta blockers, calcium channel blockers or digoxin, one study permitted choice between beta blockers or digoxin, one study allowed choice between beta blockers or calcium channel blockers, two studies used digoxin alone and one study used beta blockers alone.

### 3.2. Risk of Bias Assessment

We outline judgments about risk of bias in included studies in Supplementary Files S7–S9. Only one out of eight randomized trials reported blinding of participants and personnel and blinding of outcome assessment [20,23]. We judged risks of bias related to randomization, allocation, incomplete outcome data, and selective reporting as either low or likely low in all studies. All observational studies had serious or moderate risk of bias.

## 4. Outcomes

### 4.1. Data from Randomized Trials

**Figure 1:**
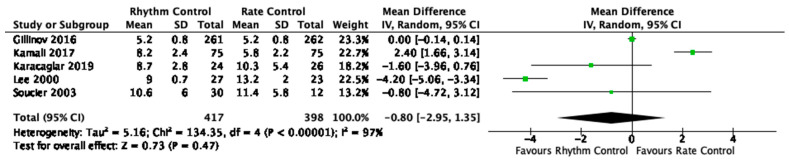
Length of stay in randomized trials. Forest plot displaying an inverse-variance weighted random-effects meta-analysis comparing rhythm and rate control on length of the index hospital stay in days (mean difference). Columns of data are displayed in the plot for all figures. The drugs and dosages in each trial are documented in Table 1 for all figures. We used studies’ definitions for clinical outcomes for all figures. The size of data markers indicates the weight of the study in all figures. Error bars indicate 95% CIs for all figures. We used RevMan 5.3 (The Cochrane Collaboration, Odense, Denmark) to combine data quantitatively for all figures [20,21,22,25,26].

Compared to rate control, rhythm control did not result in a significant reduction in length of stay (Table 2, Figure 1, Supplementary file S3). There was no statistical evidence of a subgroup effect on length of stay between studies that used amiodarone-based and non-amiodarone-based rhythm control. We rated the quality of evidence for this outcome as very low due to its skewed distribution, imprecision, inconsistency and risk of bias (Supplementary Files S7, S9, and S10).

**Figure 2:**
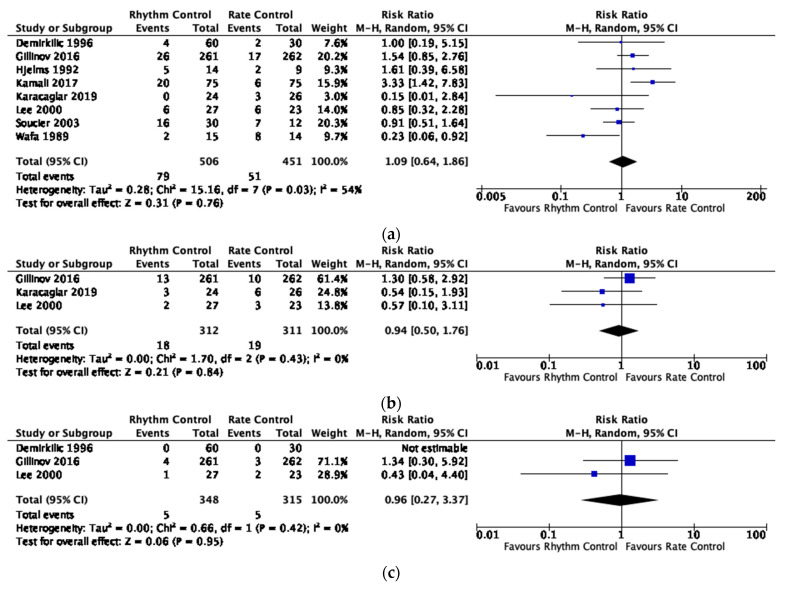
(**a**) AF recurrence within 1 week in randomized trials. Forest plot displaying relative risks calculated using a random-effects model with Mantel-Haenszel weighting comparing rhythm and rate control on atrial fibrillation recurrence within 1 week. The relative risks were calculated using a random-effects model with Mantel-Haenszel weighting for all figures [19,20,21,22,23,24,25,26]; (**b**) AF recurrence up to 1 month in randomized trials. Forest plot displaying relative risks calculated using a random-effects model with Mantel-Haenszel weighting comparing rhythm and rate control on atrial fibrillation recurrence up to 1 month [21,25,26]; (**c**) AF recurrence up to 3 months in randomized trials; Forest plot displaying relative risks calculated using a random-effects model with Mantel-Haenszel weighting comparing rhythm and rate control on atrial fibrillation recurrence up to 3 months [21,24,25].

**Figure 3:**
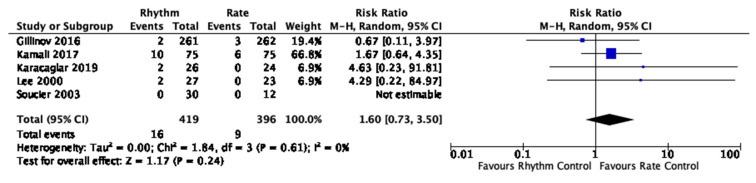
Mortality in randomized trials. Forest plot displaying relative risks calculated using a random-effects model with Mantel-Haenszel weighting comparing rhythm and rate control on mortality as defined by the respective study [20,21,22,25,26].

**Figure 4:**
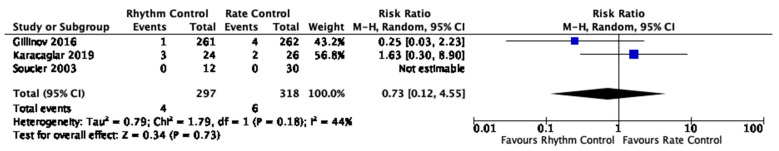
Stroke in randomized trials. Forest plot displaying relative risks calculated using a random-effects model with Mantel-Haenszel weighting comparing rhythm and rate control on stroke as defined by the respective study [22,25,26].

Compared to rate control, rhythm control did not result in a significant reduction in AF recurrence within 1 week, up to 1 month or up to 3 months, mortality or stroke (Table 3, Figure 2, Figure 3 and Figure 4, Supplementary File S3). There was no statistical evidence of a subgroup effect on mortality between studies that used amiodarone-based and non-amiodarone-based rhythm control. We rated the quality of evidence for most outcomes as low due to imprecision and risk of bias (Supplementary Files S7, S9 and S10). We rated the quality of evidence for stroke as very low due to very serious imprecision and risk of bias (Supplementary Files S7, S9 and S10).

### 4.2. Data from Observational Studies

Among observational studies, four studies reported data on length of stay, three studies reported on AF recurrence within 1 week, two studies reported on AF recurrence up to 1 month, three studies reported on AF recurrence up to 3 months and two studies reported on mortality. Effect measures from observational studies did not differ appreciably from those in randomized trials (Supplementary File S5). We rated the quality of evidence for all outcomes as very low. All outcomes were downgraded due to risk of bias. Length of stay was downgraded for non-normal distribution. All other outcomes were downgraded for serious imprecision (Supplementary File S11).

## 5. Discussion

The current literature, when synthesized, fails to demonstrate significant differences in length of stay, AF recurrence, mortality or stroke between rhythm and rate control strategies for patients with POAF after cardiac surgery. This lack of significant difference is consistent between studies that used both amiodarone-based and non-amiodarone-based rhythm control. However, this body of evidence has important limitations. The number of patients enrolled in trials evaluating rhythm and rate control strategies in postoperative atrial fibrillation is small, with fewer than 1000 participants in total. Most of these studies were open-label. Moreover, substantial variability in interventions and follow-up durations reduced confidence in estimates of effect.

To our knowledge, this systematic review and meta-analysis is the first to compare rhythm vs. rate control specifically in patients with POAF after cardiac surgery. A 2018 systematic review of RCTs comparing rhythm to rate control for patients with AF in general only included one study with POAF [34]. This meta-analysis of 12 studies showed no significant difference between rhythm and rate control groups for mortality, bleeding, and thromboembolic events but demonstrated a higher rehospitalization rate with rhythm control [34].

The largest trial in this review was conducted by the Cardiothoracic Surgical Trials Network from 2014 to 2015; it accounts for 523 of the 990 participants (52.8% of patients, 23.3% of the weight for length of stay) in the meta-analysis [25]. This trial has important limitations that deserve mention, some of which are highlighted in the 2017 EACTS Guidelines [12]. The treatment regimen of this trial included amiodarone for the rhythm control group and beta blocker/calcium channel blocker or digoxin for the rate control group. However, both groups received rate control for patients with a heart rate less than 100 and were cardioverted electrically if AF was persistent beyond 24–48 h, which may have minimized differences in treatment effect. The cross-over rate was very high (25%), and rhythm status was assessed using intermittent rather than continuous ECG. In addition, the trial included patients with short episodes of POAF—these low-risk patients may have obscured benefits seen in higher-risk patients.

As neither rhythm nor rate control is superior for the treatment of POAF in cardiac surgery patients, both strategies can be considered for the treatment of individual patients. Both the ESC and CCS guidelines suggest tailoring treatment. The 2020 ESC guidelines state that “…*rate or rhythm control treatment decisions should be based on symptoms* (Class I Recommendation, Level A Evidence)”. The 2016 CCS Guidelines state that “*choice of strategy should therefore be individualized on the basis of the degree of symptoms* (Strong Recommendation, Moderate-Quality Evidence)” [9,10]. In contrast, the 2017 EACTS guidelines state that “*In patients with postoperative haemodynamically stable POAF, rhythm control is recommended* (Class I Recommendation, Level B Evidence)” [12].

Our study suggests that large, randomized trials are required to compare rhythm and rate control for POAF in cardiac surgery patients. Future studies should assess adverse events and seek to understand clinician, economic and patient values in decision-making. This review also highlights the lack of data on other important outcomes, such as bleeding, hospital readmission, new or worsening heart failure, days out of hospital, quality of life, bleeding and thrombotic events. The International Consortium for Health Outcomes Measurement has identified these outcomes to be meaningful to both patients and clinicians and recommends them as standard outcomes for trials in AF [35,36].

## 6. Strengths and Weaknesses

Our search was comprehensive, using three large trial databases (MEDLINE, Embase, and CENTRAL) for published data, and we screened trial registries and enquired with specialists about additional studies. The review was pre-registered and used the GRADE framework to evaluate the quality of the evidence.

The principal limitations of this review are inherent to the studies that met the eligibility criteria. Variability in intervention types, follow-up periods, drug types, doses and durations, as well as a high proportion of patients lost to follow-up in the included studies, may have obscured a signal. It is worth noting that some drugs primarily intended for rhythm control, such as amiodarone, dronedarone and sotalol, can also have an impact on reducing ventricular rate during atrial fibrillation. Amiodarone, in particular, has shown effectiveness in slowing the ventricular rate in patients with atrial fibrillation and heart failure who are intolerant to high-dose β-blockade in combination with digoxin or in whom calcium channel blockers are contraindicated. However, in our review, we focused exclusively on assessing amiodarone as a rhythm control agent, and its potential role in acute heart rate control in the context of cardiac surgery was not specifically examined. Studies reported overall adverse events rather than comparative counts between rhythm and rate control, which obviated meta-analysis. Included studies ascertained AF recurrence using 12 lead-ECG and/or short-duration Holter monitoring. Implantable loop recorders (ILRs) are the most sensitive tool for detecting AF recurrence and have become increasingly used in post-ablation studies. ILRs may have led to detectable differences in AF recurrence outcomes [37]. Furthermore, since many cardiac surgery patients receive oral anticoagulation, the risk of thromboembolic events in the perioperative period overall was low, which may have affected the signal between rhythm and rate control for reducing thromboembolic events.

## 7. Conclusions

Currently, limited, low-quality data inform on the efficacy of a rhythm control vs. a rate control approach for patients with new-onset AF following cardiac surgery. A large-scale randomized trial is needed to inform this important clinical question.

## Figures and Tables

**Table 1:** Characteristics of included randomized trials.

Study ID	N	Surgery Type	Rhythm Control	Rate Control	Follow-Up Duration	Outcomes Reported
Demirkilic 1996 [24]	120	Isolated CABG: 120/120 (100%)	Choice between: -Quinidine PO 550 mg/day -Amiodarone PO 600 mg/day for 7 days then 200 mg/day	Verapamil PO 240 mg/day	1 week	AF recurrence (within 1 week, up to 1 month & up to 3 months)
Gillinov 2016 [25]	523	Isolated CABG: 212/523 (40.5%) CABG + valve repair: 17/523 (3.3%) CABG + valve replacement: 86/523 (16.4%) Non-CABG: 208/523 (39.8%)	Amiodarone 3 g PO load then 200 mg per day Both arms received rate control for HR < 100 and got DCCV if AF was persistent beyond 24–48 h	Beta blocker and/or calcium channel blocker and/or Digoxin Both arms received rate control for HR < 100 and got DCCV if AF was persistent beyond 24–48 h	60 days	Length of stay AF recurrence (within 1 week, up to 1 month & up to 3 months) Mortality Stroke
Hjelms 1992 [19]	30	Isolated CABG: 25/30 (83.3%) Non-CABG: 5/30 (16.7%)	IV Procainamide, then PO Procainamide for 1 week	Choice between: -IV Digoxin -PO Digoxin maintenance dose 0.1–0.3 mg	1 week	Length of stay AF recurrence (within 1 week) Mortality
Kamali 2017 [20]	146	Isolated CABG: 146/146 (100%)	Amiodarone PO or IV 300 mg followed by 1–3 mg/kg every 6 h and 0.5 mg/kg 18 h later	Beta blocker IV 1–3 mg/kg/h for 24 h	24 h	Length of stay AF Recurrence (within 1 week) Mortality
Karacaglar 2019 [26]	50	Isolated CABG: 43/50 (86%)CABG + valve surgery: 7/50 (14%)	IV amiodarone DCCV if in AF at 24 h then PO amiodarone for 28 days	Beta blocker, calcium channel blocker or Digoxin DCCV if in AF at 24 h	30 days	Length of stay AF Recurrence (within 1 week, up to 1 month) Bleeding Mortality Stroke
Lee 2000 [21]	50	Isolated CABG: 34/50 (68%)CABG + valve surgery: 7/50 (14%) Non-CABG: 9/50 (18%)	Choice between: -Sotalol PO 120–360 mg/day -Propafenone PO 300–900 mg/day -Procainamide IV 500–1000 mg followed by a continuous infusion of 1 to 4 mg/h or 2 to 3 g/day in divided oral doses. -Amiodarone IV 200 mg/day after a loading dose of 1200 to 1600 mg for 4 to 5 days DCCV if in AF at 48 h	Beta blocker, calcium channel blocker or Digoxin	Rhythm: 48 h Rate: Until HR ≤ 110 BPM or 110–120 BPM with no heart failure	Length of stay AF recurrence (within 1 week, up to 1 month & up to 3 months) Mortality
Soucier 2003 [22]	42	Isolated CABG: 34/42 (81%)CABG + valve surgery: 6/42 (14.3%) Non-CABG: 2/42 (4.8%)	Choice between: -IV Ibutilide -Propafenone	Physician choicebeta blocker encouraged	1 week	AF recurrence (within 1 week) Stroke
Wafa 1989 [23]	29	Isolated CABG: 29/29 (100%)	Flecainide IV for up to 24 h	IV Digoxin +/− Verapamil	24 h	Length of stay AF recurrence (within 1 week)

PO: taken orally; IV: given intravenously; AF: atrial fibrillation; N: number of randomized patients; DCCV: direct current cardioversion; CABG: coronary artery bypass graft.

**Table 2:** Summary of length of stay and subgroup analyses for the comparison of rhythm vs. rate control.

Group	N Studies (References)	Total Patients	Mean Length of Stay in Days+/− Standard Deviation		MeanDifference (95%CI)	*p*-Value	I^2n^	Quality of Evidence Reason for Judgement (Supplementary Files S7, S9a–h and S10)
			Rhythm Control	Rate Control				
All trials	5 [20,21,22,25,26]	815	6.6 ± 0.7	6.3 ± 0.7	−0.8 days (−3.0 to +1.4)	0.47	97%	Very low Skewed distribution, risk of bias, imprecision
Amiodarone-based rhythm control	3 [20,24,25]	723	6.1 ± 0.6	5.7 ± 0.6	0.5 days (−1.5 to +2.5)	0.63	95%	
Nonamiodarone-based rhythm control	2 [21,22]	92	9.8 ± 1.3	12.6 ± 1.3	−3.1 days (−6.2 to +0.1)	0.06	64%	

No significant subgroup differences for length of stay (*p* = 0.06).

**Table 3:** Summary of AF recurrence, mortality and stroke and sensitivity analyses for the comparison of rhythm vs. rate control.

Group	N Studies (References)	Number of Patients with Events/Number of Patients at Risk		Relative Risk			Quality of Evidence Reason for Judgement (Supplementary Files S7, S9 and S10)
		Rhythm Control	Rate Control	Risk Ratio (95% CI)	*p*-Value	I^2^	
	AF recurrence						
AF recurrence within one week	8 [19,20,21,22,23,24,25,26]	79/605	51/451	1.1 (0.6–1.9)	0.76	54%	Low Imprecision, risk of bias
AF recurrence up to one month	3 [21,25,26]	18/312	19/311	0.9 (0.5–1.8)	0.84	0%	
AF recurrence up to three months	3 [21,24,25]	5/348	5/315	1.0 (0.3–3.4)	0.95	0%	
	Mortality						
All studies	5 [20,21,22,25,26]	16/419	9/396	1.6 (0.7–3.5)	0.24	0%	
Amiodarone-based rhythm control	3 [20,25,26]	14/360	9/363	1.5 (0.7–3.4)	0.33	0%	Low Imprecision, risk of bias
NonAmiodarone-based rhythm control	2 [21,22]	2/57	0/35	4.3 (0.2–85.0)	0.34	N/A	
	No significant subgroup differences for mortality (*p* = 0.51)						
	Stroke						
All studies	3 [22,25,26]	4/297	6/318	0.7 (0.1–4.6)	0.73	44%	Very low Very serious imprecision, risk of bias

## Data Availability

All relevant data are within the manuscript and Supplementary Materials.

## Supplementary Materials

The following supporting information can be downloaded at: https://www.mdpi.com/article/10.3390/jcm12134534/s1.

## Abbreviations and Acronyms

Atrial fibrillation	(AF)
Canadian Cardiovascular Society	(CCS)
Coronary artery bypass graft	(CABG)
Direct current cardioversion	(DCCV)
European Society of Cardiology	(ESC)
European Association for Cardio-Thoracic Surgery	(EACTS)
Given intravenously	(IV)
GRADE	(Grading of Recommendations Assessment, Development and Evaluation)
Postoperative atrial fibrillation	(POAF)
Preferred Reporting Items for Systematic Reviews and Meta-Analyses	(PRISMA)
Taken orally	(PO)
