# Supplementary material for: Rhythm vs. Rate Control in Patients with Postoperative Atrial Fibrillation after Cardiac Surgery: A Systematic Review and Meta-Analysis
Source: J Clin Med. 2023 Jul 7;12(13):4534. doi: 10.3390/jcm12134534 (PMC10342940; doi:10.3390/jcm12134534)
Supplement: Supplementary file 1 [file jcm-12-04534-s001.zip › jcm-2418727-supplementary.pdf]

Supplementary online content

Supplementary S1 - Differences between registered protocol and final manuscript
Supplementary S2 - Search strategy
Supplementary S3 - Length of stay and mortality subgroups for randomized trials
Supplementary S4 - Study flow diagram for evidence source and selection
Supplementary S5 - Individual randomized trial rhythm monitoring method
Supplementary S6 - Characteristics and effect measures from observational studies (tables and forest plots)
Supplementary S7 - Risk of bias assessment for randomized trials
Supplementary S8 - Risk of bias assessment for observational studies
Supplementary S9 a-h - Individual risk of bias judgments for randomized trials
Supplementary S10 - Summary of findings: atrial fibrillation: rhythm vs rate control – randomized trials
Supplementary S11 - Summary of findings: atrial fibrillation: rhythm vs rate control – observational studies

This supplementary material has been provided by the authors to give readers additional information about their work.

Supplementary S1 - Differences between registered protocol and final manuscript

We could not analyze the following secondary outcomes due to a lack of study-level subgroup data:

- Bleeding
  - No events in three of the four randomized trials that reported bleeding
- Hospital readmission
  - Only one trial evaluating this outcome.
- New or worsening heart failure
  - Only one trial evaluating this outcome.
- Days out of hospital
  - No trials evaluating this outcome.
- Quality of life
  - No trials evaluating this outcome.

We were not able to conduct planned subgroup analyses for the following groups due to a lack of study-level subgroup data:

  - High and moderate vs low risk of bias studies
  - Isolated CABG vs all other surgeries

Supplementary S2 - Search strategy

CENTRAL
Date run: 18/03/2023 17:3
Inception to March 2023

ID	Search	Hits
#1	cardiac surg* or cardiac surgeon or heart surgery	44766
#2	cardiovascular surg* or cardiovascular surgery	18156
#3	atrial fibrillation or atrial fibrillation	14666
#4	afib	115
#5	atrial fib*	14890
#6	atrial flutter or heart atrium flutter	1508
#7	rhythm control	4814
#8	anti-arrythmic agent	2
#9	cardioversion or cardioversion	1658
#10	antiarrythmia or heart arrhythmia	7693
#11	amiodarone or Amiodarone	1738
#12	sotalol or Sotalol	685
#13	flecainide or Flecainide	429
#14	propafenone or propafenone	501
#15	ibutilide	95
#16	dofetilide	141
#17	disopyramide	272
#18	vernakalant	47
#19	dronedarone	150
#20	rate control	120069
#21	rate*	440409
#22	Metoprolol or metoprolol	3270
#23	Atenolol or Atenolol	3354
#24	Beta block* or beta adrenergic receptor blocking agent	14107
#25	Bisoprolol or bisoprolol	1105
#26	Adrenergic beta-antag*	4529
#27	Carvedilol or carvedilol	1494
#28	Timolol or timolol	2442
#29	non-dihydropyridine calcium channel blockers	46
#30	diltiazem	1906
#31	verapamil	2458
#32	Cardizem	34
#33	gallopamil	181
#34	Nebivolol or Nebivolol	605
#35	landiolol	220

#36	Acebutolol or Acebutolol	407
#37	Digoxin	1994
#38	cardiac glycosides	206
#39	digitoxin	101
#40	Proscillaridin	11
#41	Lanoxin	27
#42	1 or 2	1485104
#43	3 or 4 or 5 or 6	1616812
#44	7 or 8 or 9 or 10 or 11 or 12 or 13 or 14 or 15 or 16 or 17 or 18 or 19	1578945
#45	20 or 21 or 22 or 23 or 24 or 25 or 26 or 27 or 28 or 29 or 30 or 31 or 32 or 33 or 34 or 35 or 36 or 37 or 38 or 39 or 40 or 41	1906258
#46	42 and 43 and 44 and 45	6417
#47	remove duplicates from 46	965

OVID Medline
Database: OVID Medline epub ahead of print, in-process & other non-indexed citations, Ovid Medline (R) daily and Ovid Medline (R) 1946 to March 18, 2023

Date run: 18/03/2023 17:3
Inception to March 2023

ID	Search	Hits
1	Cardiac surg*.mp. or exp heart surgery/	97730
2	exp cardiovascular surgery/	611872
3	exp atrial fibrillation/	66759
4	atrial fibrillation.mp.	98212
5	afib.mp.	428
6	atrial fib*.mp.	98698
7	atrial flutter.mp.	9178
8	exp heart atrium flutter/	137442
9	exp antiarrhythmic agent/	220494
10	rhythm control.mp.	2026
11	cardioversion.mp.	6754
12	exp automatic cardioversion/ or exp cardioversion/	15641
13	heart arrhythmia.mp. or exp heart arrhythmia/	129899
14	anti-arrhythmia.mp.	28774
15	amiodarone.mp.	11610
16	exp amiodarone/	7942
17	Sotalol.mp. or exp sotalol/	3374
18	exp flecainide acetate/ or exp flecainide/ or flecainide.mp.	2680
19	propafenone.mp. or exp propafenone/	1908
20	exp ibutilide fumarate/ or exp ibutilide/ or ibutilide.mp.	397
21	dofetilide.mp. or exp dofetilide/	1052
22	exp disopyramide phosphate/ or exp disopyramide/	2096
23	vernakalant.mp. or exp vernakalant/	15674
24	exp dronedarone plus ranolazine/ or exp dronedarone/	713
25	rate control.mp.	3729
26	exp beta adrenergic receptor blocking agent/ or exp metoprolol/	86456
27	exp atenolol plus chlortalidone/ or exp atenolol/ or exp atenolol plus indapamide/ or exp atenolol plus nifedipine/	8504
28	Atenolol.mp.	1943
29	Bisoprolol.mp. or exp acetylsalicylic acid plus bisoprolol/ or exp bisoprolol/ or exp bisoprolol fumarate plus hydrochlorothiazide/ or exp bisoprolol fumarate/	41576
30	Adrenergic beta-antag*.mp.	3949
31	exp carvedilol plus ivabradine/ or exp carvedilol/	1675
32	exp dorzolamide plus timolol maleate/ or exp hydrochlorothiazide plus timolol maleate/ or exp brimonidine plus timolol/ or exp latanoprost plus	5309

	timolol/ or exp timolol plus travoprost/ or exp bimatoprost plus timolol/ or exp bendroflumethiazide plus timolol/ or exp tafluprost plus timolol/ or exp timolol maleate/ or exp brinzolamide plus timolol/ or exp timolol/ or Timolol.mp. or exp pilocarpine plus timolol/ or exp dorzolamide plus timolol/	
33	exp verapamil/ or exp calcium channel blocking agent/ or exp diltiazem/	53141
34	Cardizem.mp.	6277
35	gallopamil.mp. or exp gallopamil/	1122
36	exp hydrochlorothiazide plus nebivolol/ or exp atenolol/ or exp nebivolol/ or exp carvedilol/ or Nebivolol.mp. or nebivolol plus valsartan/	841
37	landiolol.mp. or exp landiolol/	396
38	Acebutolol.mp. or exp acebutolol plus nifedipine/ or exp acebutolol/	1147
39	exp digoxin/ or Digoxin.mp.	16876
40	cardiac glycosides.mp. or exp cardiac glycoside/	44712
41	digitoxin.mp. or exp digitoxin/	245
42	Proscillaridin.mp. or exp proscillaridin/	12603
43	Lanoxin.mp. or exp digoxin/	2971
44	1 or 2	644503
45	3 or 4 or 5 or 6 or 7 or 8	167378
46	9 or 24 or 23 or 22 or 21 or 20 or 19 or 18 or 17 or 16 or 15 or 14 or 13 or 12 or 11 or 10	357651
47	25 or 26 or 27 or 28 or 29 or 30 or 31 or 32 or 33 or 34 or 35 or 36 or 37 or 38 or 39 or 40 or 41 or 42 or 43	188660
48	44 and 45 and 46 and 47	854
49	remove duplicates from 48	856

Supplementary S3 - Length of stay and mortality subgroups for randomized trials

Length of stay subgroups for randomized trials

Study or Subgroup	Rhythm Control Mean	Rhythm Control SD	Rhythm Control Total	Rate Control Mean	Rate Control SD	Rate Control Total	Weight	Mean Difference IV, Random, 95% CI
1.6.1 Length of Stay - Amiodarone vs Rate Control								
Gillinov 2016	5.2	0.8	261	5.2	0.8	262	23.3%	0.00 [−0.14, 0.14]
Kamali 2017	8.2	2.4	75	5.8	2.2	75	22.7%	2.40 [1.66, 3.14]
Karacaglar 2019	8.7	2.8	24	10.3	5.4	26	18.2%	−1.60 [−3.96, 0.76]
Subtotal (95% CI)			360			363	64.3%	0.48 [-1.49, 2.45]
Heterogeneity: Tau² = 2.62; Chi² = 41.33, df = 2 (P < 0.00001); I² = 95%								
Test for overall effect: Z = 0.48 (P = 0.63)								
1.6.2 Length of Stay - Non-Amiodarone based Rhythm Control vs Rate Control								
Lee 2000	9	0.7	27	13.2	2	23	22.5%	−4.20 [−5.06, −3.34]
Soucier 2003	10.6	6	30	11.4	5.8	12	13.2%	−0.80 [−4.72, 3.12]
Subtotal (95% CI)			57			35	35.7%	-3.06 [-6.21, 0.09]
Heterogeneity: Tau² = 3.68; Chi² = 2.76, df = 1 (P = 0.10); I² = 64%								
Test for overall effect: Z = 1.91 (P = 0.06)								
Total (95% CI)			417			398	100.0%	-0.80 [-2.95, 1.35]
Heterogeneity: Tau² = 5.16; Chi² = 134.35, df = 4 (P < 0.00001); I² = 97%								
Test for overall effect: Z = 0.73 (P = 0.47)								
Test for subgroup differences: Chi² = 3.50, df = 1 (P = 0.06), I² = 71.4%								

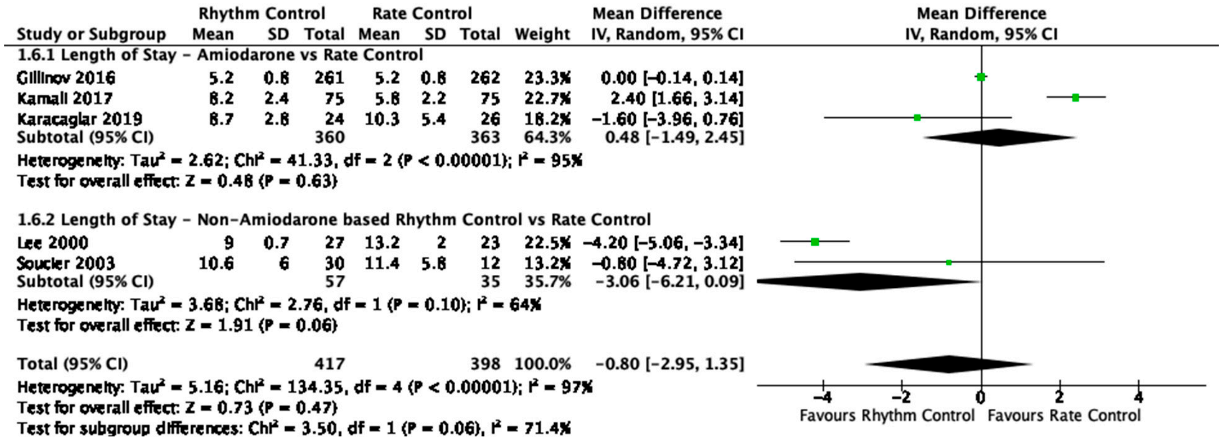

Mortality subgroups for randomized trials

Study or Subgroup	Rhythm Control Events	Rhythm Control Total	Rate Control Events	Rate Control Total	Weight	Risk Ratio M-H, Random, 95% CI
4.2.2 Mortality - Amiodarone vs. Rate Control						
Gillinov 2016	2	261	3	262	19.4%	0.67 [0.11, 3.97]
Kamali 2017	10	75	6	75	66.8%	1.67 [0.64, 4.35]
Karacaglar 2019	2	24	0	26	6.9%	5.40 [0.27, 107.09]
Subtotal (95% CI)		360		363	93.1%	1.50 [0.67, 3.39]
Total events	14		9			
Heterogeneity: Tau² = 0.00; Chi² = 1.55, df = 2 (P = 0.46); I² = 0%						
Test for overall effect: Z = 0.98 (P = 0.33)						
4.2.3 Mortality - Non-Amidarone based Rhythm Control vs. Rate Control						
Lee 2000	2	27	0	23	6.9%	4.29 [0.22, 84.97]
Soucier 2003	0	30	0	12		Not estimable
Subtotal (95% CI)		57		35	6.9%	4.29 [0.22, 84.97]
Total events	2		0			
Heterogeneity: Not applicable						
Test for overall effect: Z = 0.95 (P = 0.34)						
Total (95% CI)		417		398	100.0%	1.62 [0.74, 3.54]
Total events	16		9			
Heterogeneity: Tau² = 0.00; Chi² = 1.99, df = 3 (P = 0.57); I² = 0%						
Test for overall effect: Z = 1.20 (P = 0.23)						
Test for subgroup differences: Chi² = 0.44, df = 1 (P = 0.51), I² = 0%						

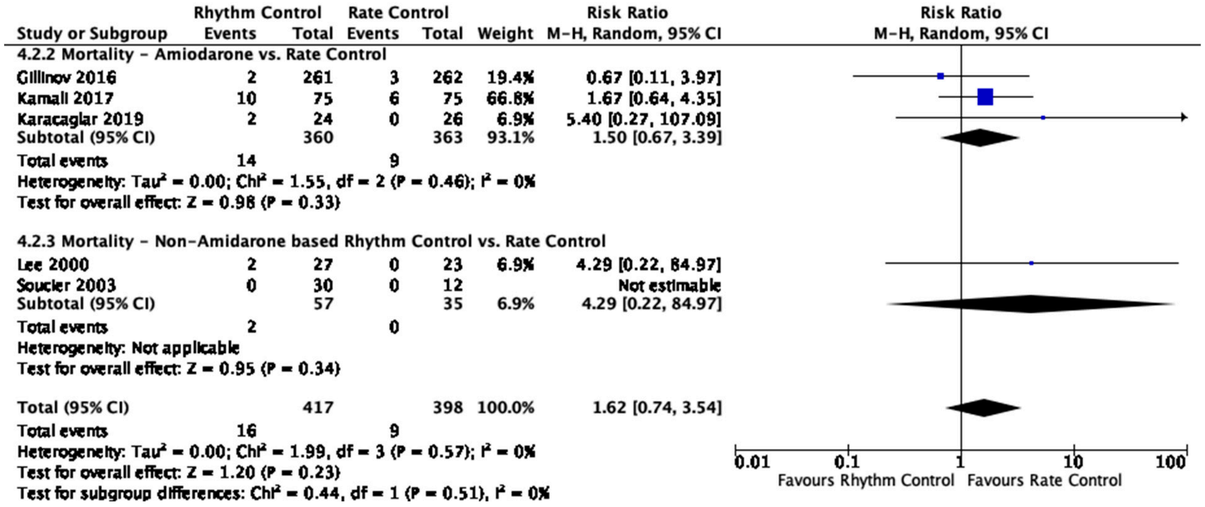

Supplementary S4 - Study flow diagram for evidence source and selection

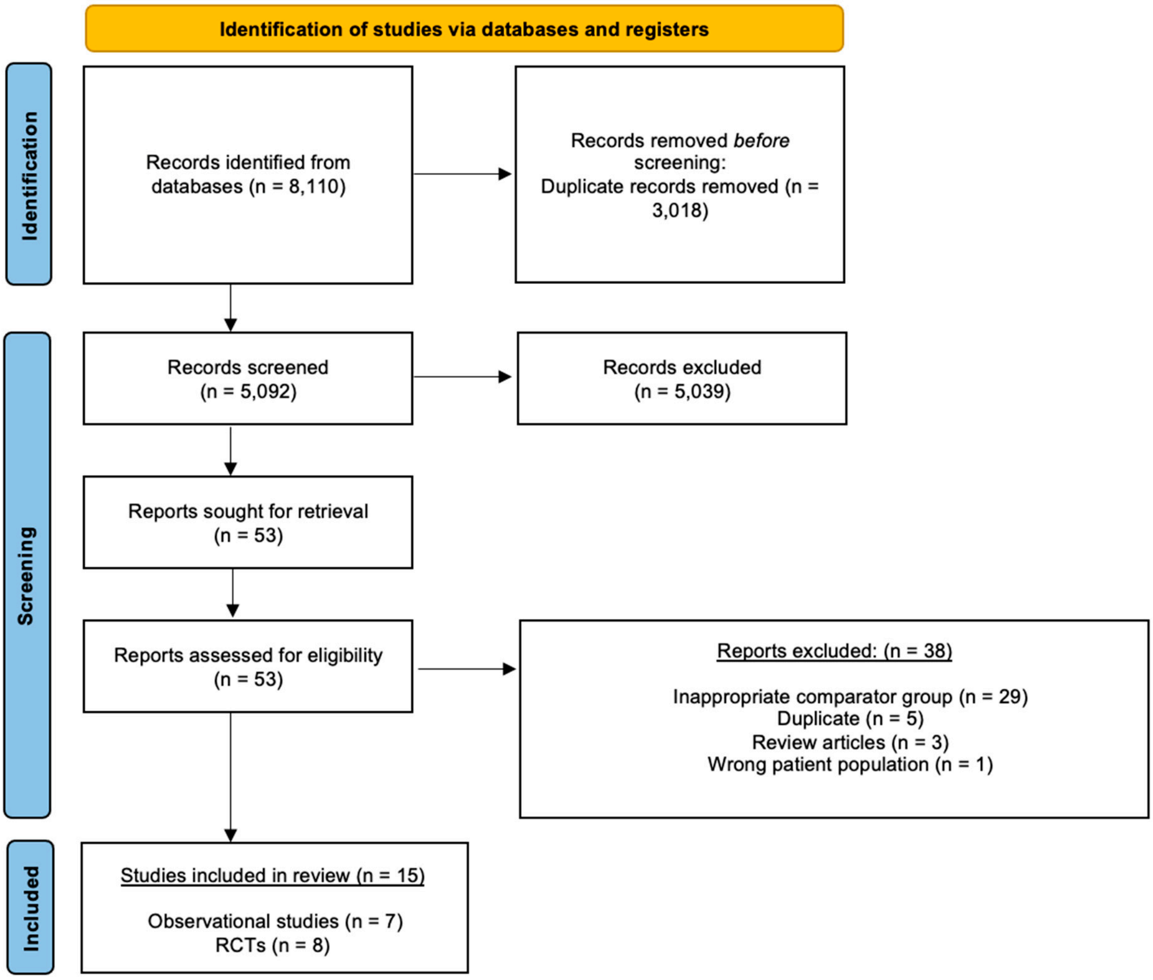

Supplementary S5 – Individual randomized trial rhythm monitoring method

Study ID	Study rhythm monitoring method
Demirkilic 1996 (17)	24-hour Holter monitoring was done weekly for 1 month and every month thereafter.
Gillinov 2016 (18)	ECG or telemetry recording was done the day of discharge, at 30 days and at 60 days.
Hjelms 1992 (12)	Unclear how long patients were followed for recurrence after initial conversion to sinus rhythm.
Kamali 2017 (13)	Rhythm monitoring not described.
Karacaglar 2019 (19)	Rhythm monitoring was done by 24-hour telemetry monitoring and confirmed with 12-lead electrocardiography during hospital follow-up. On the 30th day, a 12-lead ECG and 48 hours of Holter monitoring were performed.
Lee 2000 (14)	Continuous 3-lead electrocardiographic telemetry was performed until hospital discharge. After initial conversion, telemetry readouts were screened daily. An episode was considered significant if it lasted for >5 minutes. Daily 12-lead electrocardiograms were also obtained.<br>Electrocardiograms were obtained 1 week, 4 weeks, and 6 to 8 weeks after discharge.
Soucier 2003 (15)	All patients received telemetry monitoring for at least 24 hours following enrollment in the study. Rhythm changes including conversion to sinus, recurrence of AF, and development of other ventricular or supraventricular tachy or brady arrhythmia were recorded and placed in each patient's chart. Patients were followed until hospital discharge.
Wafa 1989 (16)	Patients were under continuous electrocardiographic monitoring throughout the 24-hour study period. On entry into the study, a 12-lead ECG electrocardiogram was taken in every patient and continuous electrocardiographic tape monitoring was started.

Supplementary S6 – Characteristics and effect measures from observational studies (tables and forest plots)

Characteristics of observational studies

**Study ID**	**N**	**Surgery type**	**Rhythm control**	**Rate control**	**Follow-up duration**	**Outcomes reported**
Abbas 2016	93	Non-CABG: 120/120 (100%)	Amiodarone IV loading dose weight (kg) x 5 (mg) and then maintenance weight (kg) x 15(mg)	Digoxin IV 0.1 mg and 0.9 BSA / 0.1	72 hours	AF recurrence (1 week)
Bruggmann 2021	97	CABG: 39/97 (40.2%)<br>Non-CABG: 58/97 (59.8%)	Amiodarone*	Choice between*<br><br>-Beta blocker<br>-Non-dihydropyridine calcium channel blocker	1 month	Length of stay<br>AF recurrence (3 months)
Cioffi 2000	176	CABG: 82/176 (46.6%)<br>Non-CABG: 94/176 (53.4%)	Amiodarone 200 mg/day	Choice between*<br><br>-Atenolol<br>-Bisoprolol<br>-Metoprolol	1 month	Length of stay<br>AF recurrence (1 month)
Doulias 2011	597	CABG: 597/597 (100%)	Amiodarone*	Choice between*<br><br>-Digoxin<br>-Beta blocker	24 hours	AF recurrence (3 months)
Guaragna 1997	101	CABG: 67/101 (66.3%)<br>Valve surgery: 35/101 (34.7%)	Amiodarone*	Digoxin*	48 hours	AF recurrence (1 week)
Shah 2001	101	CABG: 101/101 (100%)	Choice between*<br><br>-Sotalol<br>-Propafenone<br>-Procainamide	Choice between*<br><br>-Digoxin<br>-Beta blocker	1 month	Length of stay<br>AF recurrence (1 week and 1 month)<br>Mortality

			-Amiodarone<br>-Quinidine	-Non-dihydropyridine calcium channel blocker		
Stebbins 2001	263	CABG: 263 (100%)	Choice between*<br><br>-Class I antiarrhythmic<br>-Class III antiarrhythmic	Choice between*<br><br>-Digoxin<br>-Beta blocker<br>-Non-dihydropyridine calcium channel blocker	6 weeks	Length of stay<br>AF recurrence (3 months)<br>Mortality

***:** dose and route on physician preference

IV: given intravenously; **AF:** atrial fibrillation; **CABG:** coronary artery bypass graft.

Effect measures from observational studies

Study ID	N	Length of stay (days)	AF recurrence 1 week	AF recurrence 1 month	AF recurrence 3 months	Mortality
Abbas 2016[b]	93	NR	12/29 rhythm control<br>32/64 rate control	NR	NR	NR
Bruggmann 2021	97	16 ± 3.8 for rhythm control<br>16 ± 1.3 for rate control	NR	NR	49/50 on rhythm control<br>8/11 on rate control[a]	NR
Cioffi 2000[b]	176	7.9 ± 2.4 for both rhythm and rate control	NR	2/22 rhythm<br>4/42 rate	NR	NR
Doulias 2011[b]	597	NR	NR	NR	22/88 on rhythm control<br>14/48 on rate control	NR
Guaragna 1997[b]	101	NR	6/28 rhythm control<br>21/52 rate control	NR	NR	NR
Shah 2001[b]	101	8.3 on rhythm control<br>6.3 on rate control	8/62 rhythm control<br>5/39 rate control	5/62 rhythm<br>2/39 rate	NR	0/62 on rhythm control<br>1/39 on rate control
Stebbins 2001	263	10.3 ± 3.1 on rhythm control<br>9.8 ± 2.9 on rate control	NR	NR	1/36 on rhythm control<br>1/76 on rate control	0/36 on rhythm control<br>4/76 on rate control [b]

NR: not reported; **AF:** atrial fibrillation; **N:** number of randomized patients

Explanations
a. Data for patients on: Rhythm control (n =50), Rate control (n =11), None (n =8), Mixed (n =28)
b. Insufficient information to calculate standard deviation

Length of stay (days) – observational studies

Study or Subgroup	Rhythm Control Mean	Rhythm Control SD	Rhythm Control Total	Rate Control Mean	Rate Control SD	Rate Control Total	Weight	Mean Difference IV, Random, 95% CI
Bruggmann 2021	16	3.8	50	16	1.3	11	26.3%	0.00 [−1.30, 1.30]
Cioffi 2000	7.9	2.4	62	7.9	2.4	42	50.5%	0.00 [−0.94, 0.94]
Shah 2001	8.3	0	62	6.3	0	39		Not estimable
Stebbins 2001	10.3	3.1	36	9.8	2.9	36	23.2%	0.50 [−0.89, 1.89]
Total (95% CI)			210			128	100.0%	0.12 [−0.55, 0.78]

Heterogeneity: Tau² = 0.00; Chi² = 0.38, df = 2 (P = 0.83); I² = 0%
Test for overall effect: Z = 0.34 (P = 0.73)

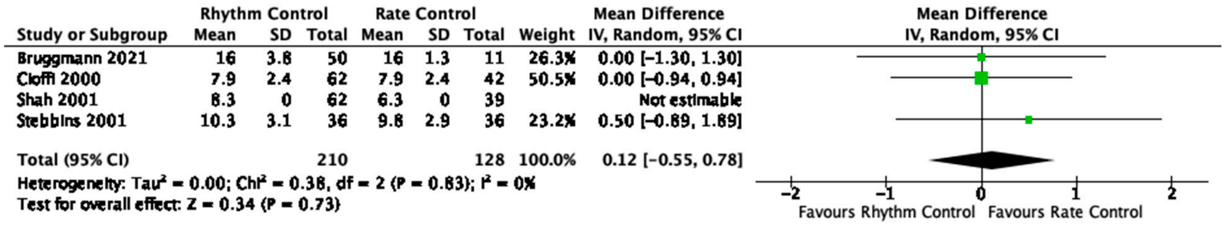

Shah 2001 did not provide information on variance, including no information on minimum, median or max. Consequently, SD was not derived.

AF recurrence (within 1 week) – observational studies

Study or Subgroup	Rhythm Control Events	Rhythm Control Total	Rate Control Events	Rate Control Total	Weight	Risk Ratio M-H, Random, 95% CI
Abbas 2016	12	29	32	64	61.3%	0.83 [0.50, 1.36]
Guaragna 1997	6	28	21	52	24.8%	0.53 [0.24, 1.16]
Shah 2001	8	62	5	39	13.9%	1.01 [0.35, 2.86]
Total (95% CI)		119		155	100.0%	0.76 [0.52, 1.12]
Total events	26		58			

Heterogeneity: Tau² = 0.00; Chi² = 1.21, df = 2 (P = 0.55); I² = 0%
Test for overall effect: Z = 1.37 (P = 0.17)

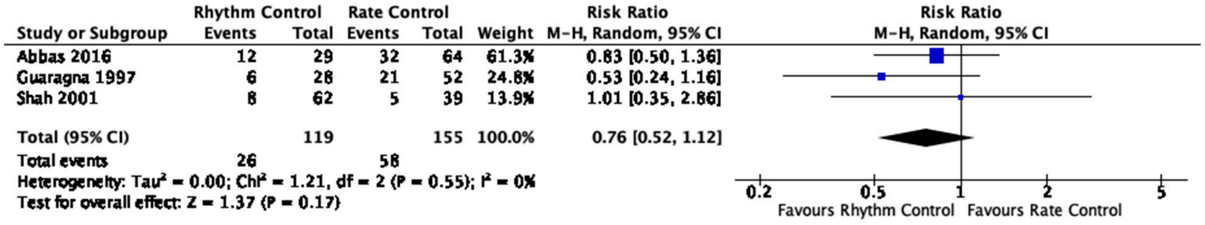

AF recurrence (up to 1 month) – observational studies

Study or Subgroup	Rhythm Control Events	Rhythm Control Total	Rate Control Events	Rate Control Total	Weight	Risk Ratio M-H, Random, 95% CI
Cioffi 2000	2	22	4	42	49.2%	0.95 [0.19, 4.81]
Shah 2001	5	62	2	39	50.8%	1.57 [0.32, 7.71]
Total (95% CI)		84		81	100.0%	1.23 [0.40, 3.82]
Total events	7		6			

Heterogeneity: Tau² = 0.00; Chi² = 0.19, df = 1 (P = 0.67); I² = 0%
Test for overall effect: Z = 0.36 (P = 0.72)

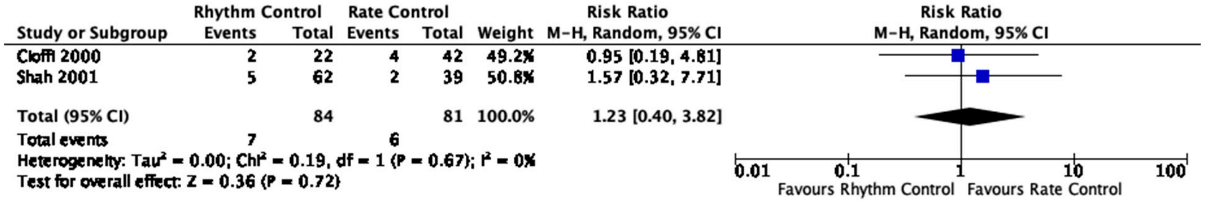

AF recurrence (up to 3 months) – observational studies

Study or Subgroup	Rhythm Control Events	Rhythm Control Total	Rate Control Events	Rate Control Total	Weight	Risk Ratio M-H, Random, 95% CI
Bruggmann 2021	49	50	8	11	66.7%	1.35 [0.94, 1.94]
Doulias 2011	22	88	14	48	31.7%	0.86 [0.48, 1.52]
Stebbins 2001	1	36	1	36	1.6%	1.00 [0.07, 15.38]
Total (95% CI)		174		95	100.0%	1.16 [0.83, 1.64]
Total events	72		23			

Heterogeneity: $Tau^2 = 0.01$; $Chi^2 = 2.19$, df = 2 (P = 0.33); $I^2 = 9\%$
Test for overall effect: Z = 0.86 (P = 0.39)

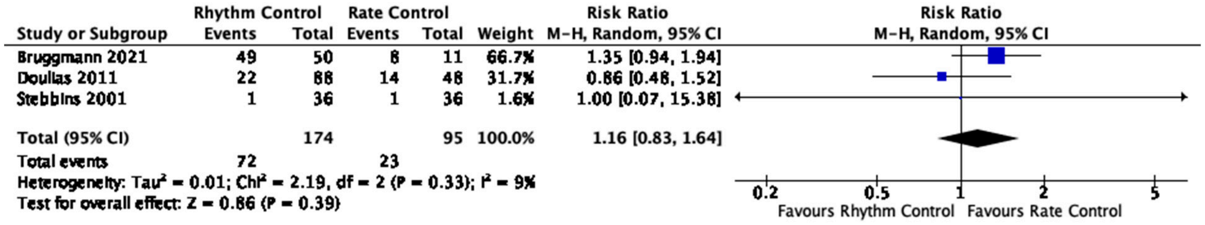

Mortality – observational studies

Study or Subgroup	Rhythm Control Events	Rhythm Control Total	Rate Control Events	Rate Control Total	Weight	Risk Ratio M-H, Random, 95% CI
Shah 2001	0	62	1	39	45.4%	0.21 [0.01, 5.07]
Stebbins 2001	0	36	4	76	54.6%	0.23 [0.01, 4.18]
Total (95% CI)		98		115	100.0%	0.22 [0.03, 1.89]
Total events	0		5			

Heterogeneity: $Tau^2 = 0.00$; $Chi^2 = 0.00$, df = 1 (P = 0.97); $I^2 = 0\%$
Test for overall effect: Z = 1.38 (P = 0.17)

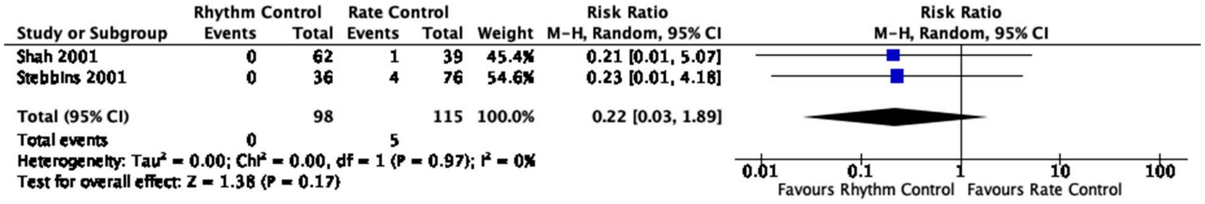

Supplementary S7 - Risk of bias assessment for randomized trials

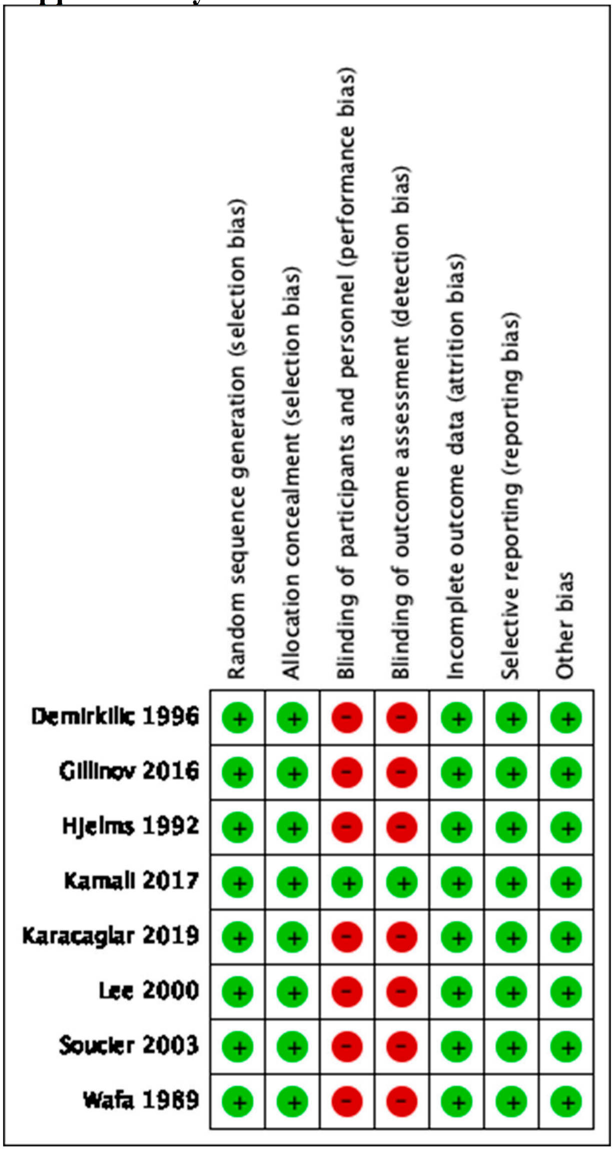

Green: low risk of bias, yellow: unclear risk of bias, red: high risk of bias

Supplementary S8 - Risk of bias assessment for observational studies

Risk of bias domains

Study	D1	D2	D3	D4	D5	D6	D7	Overall
Abbas 2016	!	+	+	-	+	+	-	X
Bruggmann 2021	!	+	+	+	+	!	+	-
Cioffi 2000	+	+	+	+	-	-	-	-
Doulias 2011	X	+	!	X	X	+	X	X
Guaragna 1997	+	+	!	-	+	+	+	-
Shah 2001	!	+	+	!	+	!	+	X
Stebbins 2001	-	+	!	!	+	+	-	-

Domains:
D1: Bias due to confounding.
D2: Bias due to selection of participants.
D3: Bias in classification of interventions.
D4: Bias due to deviations from intended interventions.
D5: Bias due to missing data.
D6: Bias in measurement of outcomes.
D7: Bias in selection of the reported result.

Judgement
! Critical
X Serious
- Moderate
+ Low

Supplementary S9 a-h - Individual risk of bias judgments for randomized trials

Supplementary S9 a) Demirkilic 1996

Risk of bias

**Bias domain**	**Judgment**	**Support for judgment**
**Random sequence generation**<br><br>**(Selection bias)**	Low	No concerns
**Allocation concealment (Selection bias)**	Unclear, Likely Low	No information, but no obvious imbalances in baseline characteristics between groups
**Blinding of participants, personnel, and outcome assessment**<br><br>**(Detection bias)**	High	No mention of blinding
**Incomplete outcome data**<br><br>**(Attrition bias)**<br><br>***All outcomes***	Low	No concerns
**Selective reporting (Reporting bias)**<br><br>***All outcomes***	Low	No concerns
**Other bias**	Low	No concerns

**Supplementary S9 b)** Gillinov 2016		
**Risk of bias**		
**Bias domain**	**Judgment**	**Support for judgment**
**Random sequence generation**<br><br>**(Selection bias)**	Low	No concerns
**Allocation concealment (Selection bias)**	Low	Randomly permuted blocks of 2, 4, 6, stratified by center
**Blinding of participants, personnel, and outcome assessment**<br><br>**(Detection bias)**	High	No mention of blinding
**Incomplete outcome data**<br><br>**(Attrition bias)**<br><br>***All outcomes***	Low	No concerns
**Selective reporting (Reporting bias)**<br><br>***All outcomes***	Low	No concerns
**Other bias**	Low	No concerns

**Supplementary S9 c)** Hjelms 1992		
**Risk of bias**		
**Bias domain**	**Judgment**	**Support for judgment**
**Random sequence generation**<br><br>**(Selection bias)**	Likely Low	No information, but no obvious imbalances in baseline characteristics between groups
**Allocation concealment (Selection bias)**	Likely Low	No information, but no obvious imbalances in baseline characteristics between groups
**Blinding of participants, personnel, and outcome assessment**<br><br>**(Detection bias)**	High	No mention of blinding
**Incomplete outcome data**<br><br>**(Attrition bias)**<br><br>***All outcomes***	Low	No concerns
**Selective reporting (Reporting bias)**<br><br>***All outcomes***	Low	No concerns
**Other bias**	Low	No concerns

**Supplementary S9 d)** Kamali 2017		
**Risk of bias**		
**Bias domain**	**Judgment**	**Support for judgment**
**Random sequence generation**<br><br>**(Selection bias)**	Low	No concerns
**Allocation concealment (Selection bias)**	Likely Low	No information, but no obvious imbalances in baseline characteristics between groups
**Blinding of participants, personnel, and outcome assessment**<br><br>**(Detection bias)**	Low	No concerns
**Incomplete outcome data**<br><br>**(Attrition bias)**<br><br>***All outcomes***	Low	No concerns
**Selective reporting (Reporting bias)**<br><br>***All outcomes***	Low	No concerns
**Other bias**	Low	No concerns

**Supplementary S9 e)** Karacaglar 2019		
**Risk of bias**		
**Bias domain**	**Judgment**	**Support for judgment**
**Random sequence generation**<br><br>**(Selection bias)**	Low	No concerns
**Allocation concealment (Selection bias)**	Likely Low	No information, but no obvious imbalances in baseline characteristics between groups
**Blinding of participants, personnel, and outcome assessment**<br><br>**(Detection bias)**	High	Study mentions that it is "open-label"
**Incomplete outcome data**<br><br>**(Attrition bias)**<br><br>***All outcomes***	Low	No loss to follow-up
**Selective reporting (Reporting bias)**<br><br>***All outcomes***	Low	No evidence
**Other bias**	Low	No evidence

**Supplementary S9 f)** Lee 2000		
**Risk of bias**		
**Bias domain**	**Judgment**	**Support for judgment**
**Random sequence generation**<br><br>**(Selection bias)**	Low	No concerns
**Allocation concealment (Selection bias)**	Likely Low	No information, but no obvious imbalances in baseline characteristics between groups
**Blinding of participants, personnel, and outcome assessment**<br><br>**(Detection bias)**	High	Study mentions that it is "unblinded"
**Incomplete outcome data**<br><br>**(Attrition bias)**<br><br>***All outcomes***	Likely Low	No mention of attrition, other than 2 patients who died, both in the rhythm group
**Selective reporting (Reporting bias)**<br><br>***All outcomes***	Low	Followed approved study protocol; researchers report pre-specified outcome measures
**Other bias**	Low	No evidence

**Supplementary S9 g)** Soucier 2003		
**Risk of bias**		
**Bias domain**	**Judgment**	**Support for judgment**
**Random sequence generation**<br><br>**(Selection bias)**	Likely Low	No information, but no obvious imbalances in baseline characteristics between groups
**Allocation concealment (Selection bias)**	Likely Low	No information, but no obvious imbalances in baseline characteristics between groups
**Blinding of participants, personnel, and outcome assessment**<br><br>**(Detection bias)**	High	Study mentions that it is "open-label"
**Incomplete outcome data**<br><br>**(Attrition bias)**<br><br>***All outcomes***	Low	No loss to follow-up
**Selective reporting (Reporting bias)**<br><br>***All outcomes***	Low	No evidence
**Other bias**	Low	No evidence

**Supplementary S9 h)** Wafa 1989		
**Risk of bias**		
**Bias domain**	**Judgment**	**Support for judgment**
**Random sequence generation**<br><br>**(Selection bias)**	Low	Specific instructions regarding randomization were reported
**Allocation concealment (Selection bias)**	Low	Sealed envelope used for allocation
**Blinding of participants, personnel, and outcome assessment**<br><br>**(Detection bias)**	High	Did not state
**Incomplete outcome data**<br><br>**(Attrition bias)**<br><br>***All outcomes***	Low	No loss to follow-up
**Selective reporting (Reporting bias)**<br><br>***All outcomes***	Low	No evidence
**Other bias**	Low	No evidence

Supplementary S10 - Summary of findings: atrial fibrillation: rhythm vs rate control – randomized trials

Certainty assessment							№ of patients		Effect		Certainty
№ of studies	Study design	Risk of bias	Inconsistency	Indirectness	Imprecision	Other considerations	Rhythm control	Rate control	Relative (95% CI)	Absolute (95% CI)	
**Length of stay**											
5	randomized trials	serious[a]	serious[b and c]	not serious	serious[d]	skewed distribution change throughout data	417	398	-	MD **0.8 day lower** (3.0 lower to 1.4 higher)	⊕◯◯◯ Very low
**Atrial fibrillation recurrence within 1 week**											
8	randomized trials	serious[a]	not serious	not serious	serious[d]	none	79/506 (15.6%)	51/451 (11.3%)	**RR 1.1** (0.6 to 1.9)	**10 more per 1,000** (from 41 fewer to 97 more)	⊕⊕◯◯ Low
**Atrial fibrillation recurrence up to 1 month**											
3	randomized trials	serious[a]	not serious	not serious	serious[d]	none	18/312 (5.8%)	19/311 (6.1%)	**RR 0.9** (0.5 to 1.8)	**4 fewer per 1,000** (from 31 fewer to 46 more)	⊕⊕◯◯ Low
**Atrial fibrillation recurrence up to 3 months**											
3	randomized trials	serious[a]	not serious	not serious	serious[d]	none	5/348 (1.4%)	5/315 (1.6%)	**RR 1.0** (0.3 to 3.4)	**1 fewer per 1,000** (from 12 fewer to 38 more)	⊕⊕◯◯ Low

Mortality

5	randomized trials	serious[a]	not serious	not serious	serious[d]	none	16/419 (3.8%)	9/396 (2.3%)	**RR 1.6** (0.7 to 3.5)	**14 more per 1,000** (from 6 fewer to 57 more)	⊕⊕◯◯ Low

Stroke

3	randomized trials	serious[a]	not serious	not serious	very serious[e]	none	4/297 (1.3%)	6/318 (1.9%)	**RR 0.7** (0.1 to 4.6)	**2 fewer per 1,000** (from 7 fewer to 29 more)	⊕◯◯◯ Very low

CI: confidence interval; **MD:** mean difference; **RR:** risk ratio

Explanations

a. Serious concerns with blinding
b. High heterogeneity
c. Certainty of evidence was not downgraded for inconsistency because it was already downgraded for imprecision
d. Wide confidence interval
e. Very wide confidence interval

Supplementary S11 - Summary of findings: atrial fibrillation: rhythm vs rate control – observational studies

Certainty assessment							№ of patients		Effect		Certainty
№ of studies	Study design	Risk of bias	Inconsistency	Indirectness	Imprecision	Large effect, dose-response effect, all plausible confounding and bias	Rhythm	Rate	Relative (95% CI)	Absolute (95% CI)	
**Length of stay**											
4	observational studies	serious[a]	not serious	not serious	not serious	yes[b]	210	128	-	MD **0.1 day higher** (0.6 lower to 0.8 higher)	⊕◯◯◯ Very low
**Atrial fibrillation recurrence within 1 week**											
3	observational studies	serious[a]	not serious	not serious	serious[c]	none	26/119 (21.9%)	56/155 (36.1%)	**RR 0.8** (0.5 to 1.1)	**3 fewer per 1,000** (from 5 fewer to 1 more)	⊕◯◯◯ Very low
**Atrial fibrillation recurrence up to 1 month**											
2	observational studies	serious[a]	not serious	not serious	serious[c]	none	7/84 (8.3%)	6/81(7.4%)	**RR 1.2** (0.4 to 3.8)	**2 more per 1,000** (from 5 fewer to 26 more)	⊕◯◯◯ Very low

Certainty assessment							№ of patients		Effect		Certainty
№ of studies	Study design	Risk of bias	Inconsistency	Indirectness	Imprecision	Large effect, dose-response effect, all plausible confounding and bias	Rhythm	Rate	Relative (95% CI)	Absolute (95% CI)	
**Atrial fibrillation recurrence up to 3 months**											
3	observational studies	serious[a]	not serious	not serious	serious[c]	none	72/174 (41.4%)	23/95 (24.2%)	**RR 1.2** (0.8 to 1.6)	**8 more per 1,000** (from 6 fewer to 108 more)	⊕◯◯◯ Very low
**Mortality**											
2	observational studies	serious[a]	not serious	not serious	serious[c]	none	0/98 (0%)	5/115 (4.3%)	**RR 0.2** (0 to 1.9)	**6 fewer per 1,000** (from 8 fewer to 7 more)	⊕◯◯◯ Very low

CI: confidence interval; **MD:** mean difference; **RR:** risk ratio

Explanations

a. Moderate risk of bias issues in all observational studies
b. Non-normal distribution
c. Wide confidence intervals
